# Diversity of HIV-1 Subtype B: Implications to the Origin of BF Recombinants

**DOI:** 10.1371/journal.pone.0011833

**Published:** 2010-07-28

**Authors:** Élcio Leal, Fabiola E. Villanova

**Affiliations:** Federal University of Pará, Belém, Brazil; Institute of Infectious Disease and Molecular Medicine, South Africa

## Abstract

**Background:**

The HIV-1 subtype B epidemic in Brazil is peculiar because of the high frequency of isolates having the GWGR tetramer at V3 loop region. It has been suggested that GWGR is a distinct variant and less pathogenic than other subtype B isolates.

**Methodology/Principal Findings:**

Ninety-four percent of the HIV-1 subtype B worldwide sequences (7689/8131) obtained from the Los Alamos HIV database contain proline at the tetramer of the V3 loop of the *env* gene (GPGR) and only 0.74% (60/8131) have tryptophan (GWGR). By contrast, 48.4% (161/333) of subtype B isolates from Brazil have proline, 30.6% (102/333) contain tryptophan and 10.5% (35/333) have phenylalanine (F) at the second position of the V3 loop tip. The proportion of tryptophan and phenylalanine in Brazilian isolates is much higher than in worldwide subtype B sequences (chi-square test, *p* = 0.0001). The combined proportion of proline, tryptophan and phenylalanine (GPGR+GWGR+GFGR) of Brazilian isolates corresponds to 89% of all amino acids in the V3 loop. Phylogenetic analysis revealed that almost all subtype B isolates in Brazil have a common origin regardless of their motif (GWGR, GPGR, GGGR, etc.) at the V3 tetramer. This shared ancestral origin was also observed in CRF28_BF and CRF29_BF in a genome region (free of recombination) derived from parental subtype B. These results imply that tryptophan substitution (*e.g*., GWGR-to-GxGR), which was previously associated with the change in the coreceptor usage within the host, also occurs at the population level.

**Conclusions/Significance:**

Based on the current findings and previous study showing that tryptophan and phenylalanine in the V3 loop are related with coreceptor usage, we propose that tryptophan and phenylalanine in subtype B isolates in Brazil are kept by selective mechanisms due to the distinct coreceptor preferences in target cells of GWGR, GFGR and GFGR viruses.

## Introduction

HIV-1 has extraordinary potential to generate diversity due to the elevated error incorporation rate of viral reverse transcriptase during the numerous replication events in infected patients. At the population level, this diversity is observed by the presence of distinct groups (M, N, and O) that evolved independently after cross-species transmission [1], [2], [3]. Group M can be classified into distinct lineages (subtypes A, B, C, D, F, G, H, J, and K), which present distinctive biological properties, including differences in their adaptive evolution [4], [5], rate of neutral mutations [6], acquisition of antiretroviral resistance [7] and tropism during cell culture [8]. Subtype B, which is involved in the AIDS epidemic worldwide and is the main source of HIV-1 infection in European and American countries [1], [9], [10], [11], [12], [13], exhibits a certain degree of genetic variability. Isolates from Thailand are remarkably distinguishable from other subtype B isolates in phylogenetic trees [10]. In Brazil, subtype B infections are characterized by high frequencies of viruses with the unusual GWGR motif in the V3 loop of the *env* gene [10], [14], [15], [16], [17]. Although GWGR viruses have occasionally been observed in other countries, such as China, France, the Czech Republic, the Philippines, and Cuba, high frequencies of GWGR isolates are seen only in Brazil. However, previous studies showed that is not possible to distinguish Brazilian isolates with the GWGR motif from those with GPGR based on phylogenetic trees from the V3 loop sequence [10], [14], [17].

We constructed phylogenies of near full-length HIV-1 genomes and partial *env* gene sequences to show that almost all isolates of the subtype B lineage in Brazil have a common origin, regardless of the tetramer sequence (such as GWGR, GPGR, GFGR) in the V3 loop.

## Results

### Tryptophan frequency

A sequence analysis of the V3 loop of subtype B viruses collected from Los Alamos HIV database was performed, revealing that 94.55% (7689/8131) of subtype B isolates worldwide have proline (P) at the tip of the V3 loop whereas only 0.74% (60/8131) have tryptophan (W) (Table 1). By contrast, 48.4% (161/333) of subtype B isolates from Brazil have proline and 30.6% (102/333) contain tryptophan and 10.5% (35/333) contain phenylalanine. Notably, the proportion of tryptophan and phenylalanine in Brazilian isolates is much higher than in worldwide subtype B sequences (chi-square test, *p* = 0.0001) whereas the combined proportion of proline, tryptophan and phenylalanine (GPGR+GWGR+GFGR) of Brazilian isolates is similar to that of only proline (GPGR) in subtype B isolates worldwide (chi-square test, *p* = 0.89).

**Table 1:** Amino acids frequency at the second position of the GxGR tetramer of V3 loop of HIV-1 subtype B.

Amino Acid	Pandemic Isolates (n = 8131)*	Brazilian Isolates (n = 333)
P	7689 (94.55%)	161 (48.4%)
L	202 (2.5%)	19 (5.7%)
W	60 (0.74%)	102 (30.6%)
Q	66 (0.81%)	2 (0.6%)
F	12 (0.15%)	35 (10.5%)
M	22 (0.27%)	3 (0.9%)
R	22 (0.27%)	3 (0.9%)
others	58 (0.71%)	8 (2.4%)

*) Sequences from worldwide countries. All sequences were obtained from the Los Alamos HIV-1 database (http://www.hiv.lanl.gov/).

P = proline; L = leucine; W = tryptophan; Q = glutamine;

F = phenylalanine; M = Methionine; R = Arginine.

Others =  alanine, lysine, serine, glycine, valine, histidine, threonine and tyrosine.

### Near full-length trees of subtype B

**Figure 1:**
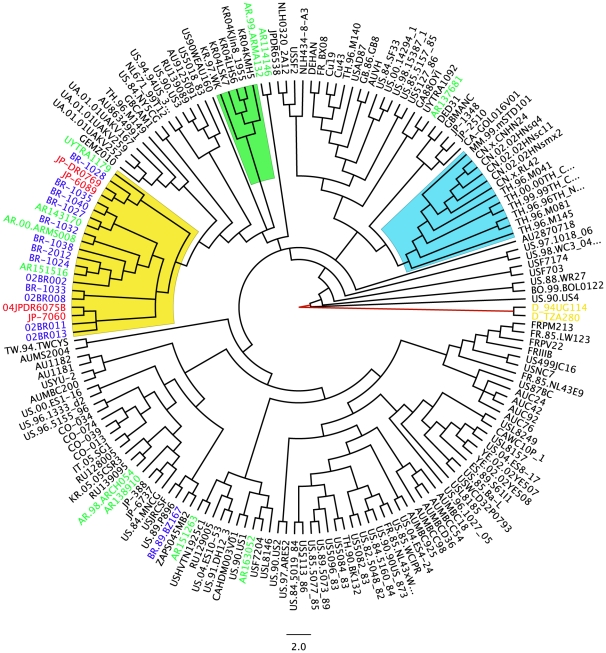
Near full-length HIV-1 Bayesian tree. The tree was constructed using 180 near full-length genomes (8487-bp) of worldwide HIV-1 isolates. The tree was converted into a cladogram and the branches are colored to indicate the a posteriori support values of the node support, and red indicates values higher than 0.7. Subtype D (TZA20 and 94UG114) sequences were used as the outgroup (yellow letters). Brazilian, Japanese, and Argentinean isolates are indicated in blue, red and green letters, respectively. The yellow area corresponds to the cluster formed with the Brazilian isolates. The green area delineates the cluster with the Korean sequences and the cyan area indicates the cluster formed with the Chinese and Thai sequences. The geographical location of the sequences are identified as follows: BR = Brazil, AR = Argentina, UY = Uruguay, CO = Colombia, AU = Australia, JP = Japan, TH = Thailand, CH = China, JP = Japan, HT = Haiti, TT =  Trinidad & Tobago.

**Figure 2:**
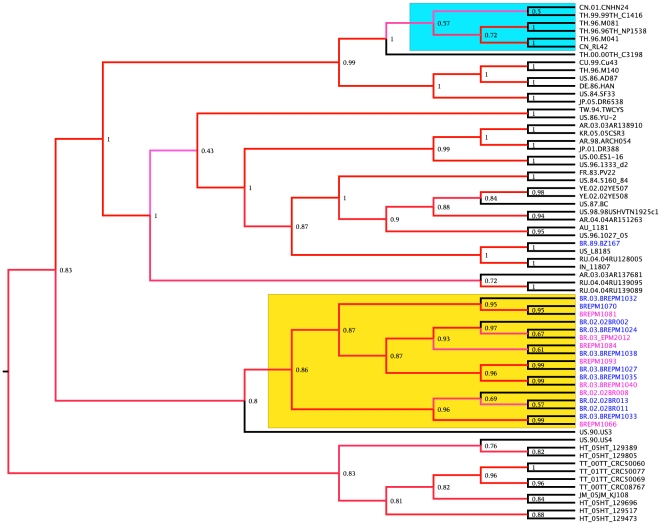
Coalescence near full-length HIV-1 Bayesian tree. A Bayesian coalescent tree was constructed using 8160-bp sequences of HIV-1 subtype B. Sequences of 66 worldwide isolates were collected from the Los Alamos HIV sequence database. The yellow area delineates the cluster formed with the Brazilian isolates and the cyan area depicts the cluster formed with the Chinese and Thai sequences. The Brazilian isolates are indicated in blue (GxGR viruses) and in magenta (GWGR viruses) in the terminal branches of the tree. Numbers in the nodes indicate the a posteriori support values. This tree was summarized from a sample of trees generated by the BMCMC run (see Material and Methods for details).

Four hundred near full-length genomes from subtype B HIV-1 were initially used to construct a maximum likelihood tree, which allowed us to filter sequences for further analysis (tree not shown). We observed that most European and North American subtype B isolates are not phylogenetically distinguishable. Next, Bayesian maximum posteriori trees were constructed with near full-length genomes of HIV-1. The topology of these trees showed little depth of the internal branches but revealed the presence of some clades composed by geographical isolates. A tree constructed with 180 near full-length genomes is depicted as a cladogram in Figure 1 and shows that the worldwide sequences are evenly dispersed. The posterior probability support for the nodes is represented by colors (*viz*. from zero to one, which are represented by a color gradient from white to red in the nodes of the tree). The European and North American isolates are also completely mixed in the tree. There is weak geographical structure in the phylogeny of the subtype B lineage of HIV-1, but some geographical clusters with very high maximum posteriori support are also present (highlighted in Figure 1). Some isolates from Thailand, South China and Myanmar form a well-supported clade (cyan area in Figure 1). In addition, some isolates from South Korea are also grouped in a unique cluster (green area, Figure 1) and all of the Brazilian isolates, except the sequence BZ167 (AY173956) that was isolated in the 1990 from PBMC cultured, form a cluster that includes some Japanese and Argentinean isolates (yellow area, Figure 1). To better illustrate the ancestral relationship of Brazilian isolates, a coalescent tree was inferred using only 66 sequences of subtype B lineage free of recombination. The coalescent tree contains all of the Brazilian isolates in a single cluster, except for isolate BZ167, with a high posteriori probability of 0.86 supporting the cluster. Interestingly, isolates with the GPGR motif (blue letters, Figure 2) and those with GWGR (pink letters, Figure 2) are equally distributed in this cluster. The trees constructed with the available near full-length genomes of subtype B from Brazil suggest that this lineage is monophyletic. In a previous study, we used eight near full-length subtype B isolates to show that GWGR viruses are not separated from other subtype B isolates in Brazil [14]. Here, we extended the analysis to show that subtype B in Brazil is likely monophyletic with most isolates sharing the same ancestor sequence. Notably, isolate BZ167 was not included in the Brazilian cluster. This isolate was one of the first viruses to be sequenced in 1989 in Brazil and may not be representative of HIV-1 in this country. Indeed, phylogenies indicated that Brazilian isolates share a common ancestor with a sequence (US3) isolated in 1990 in the United States. Interestingly, some Japanese isolates also clustered with Brazilian isolates, likely because the high migration flux between these countries contributed to cross HIV infection. Another important feature observed in the genome trees was the distribution pattern of Argentinean subtype B isolates. Some Argentinean isolates were found within the Brazilian cluster and likely represent cross infections between these neighboring countries. Most isolates from Argentina were evenly spread in the near full-length topologies (green letters, Figure 1), suggesting that the subtype B isolates in that country consist of multiple and distinct sources of infection. By contrast, subtype B in Brazil possibly originated from fewer HIV-1 sources. Furthermore, most sequences from Thailand, South China, Korea and the Caribbean form well-defined clusters, whereas little geographical structure was observed among isolates from Europe and North America (Figure 1).

### Phylogenetic relatedness of CRF_BF

**Figure 3:**
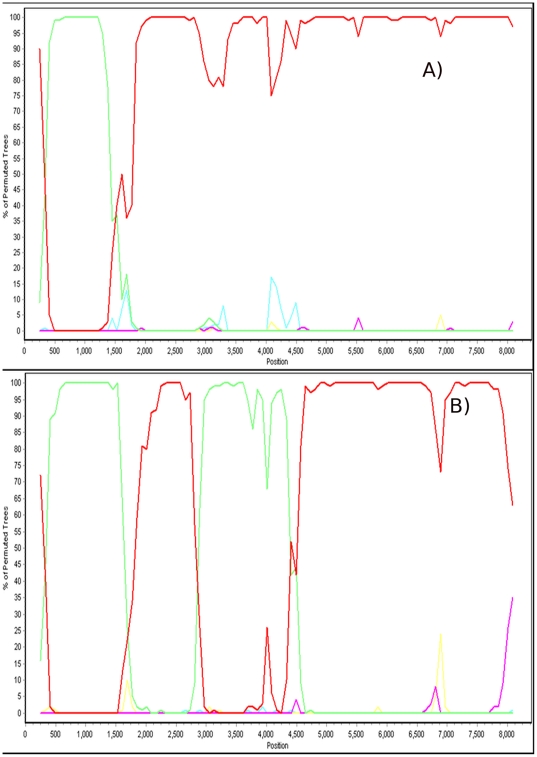
Mosaic pattern of HIV-1 CRF_BF in Brazil. Panel A depicts the recombination pattern of CRF_28BF isolates (12609, 12817 and 12313) and panel B depicts the recombination pattern of the CRF_29BF isolates (11948, 16704 and 99ufrj1). The x-axis shows the sequence length in base pairs (bp). The y-axis displays the bootstrap support based on 100 replicates. Red line corresponds to genome regions of CRF_BF related with the subtype B lineage and green line correspond to regions related with the subtype F lineage. Analyses were performed using a neighbor-joining method and the Kimura two-parameter model in 500-bp windows sliding along sequences in increments of 80 bp. The GenBank accession numbers of the non-recombinant sequences used as parental references are: subtype A (AF004885), subtype B (AY423387, M38432, and AY331295), subtype D (K03454, AY253311, and U88824), subtype G (AF084936 and AF061642), subtype J (AF082394), subtype K (AJ249235 and AJ249239), subtype C (U52953, AF067155, and AY772699), subtype H (AF005496) and subtype F (AF005494). The bootscan analysis was performed with Simplot v.2.5 (http://sray.med.som.jhmi.edu/RaySoft).

**Figure 4:**
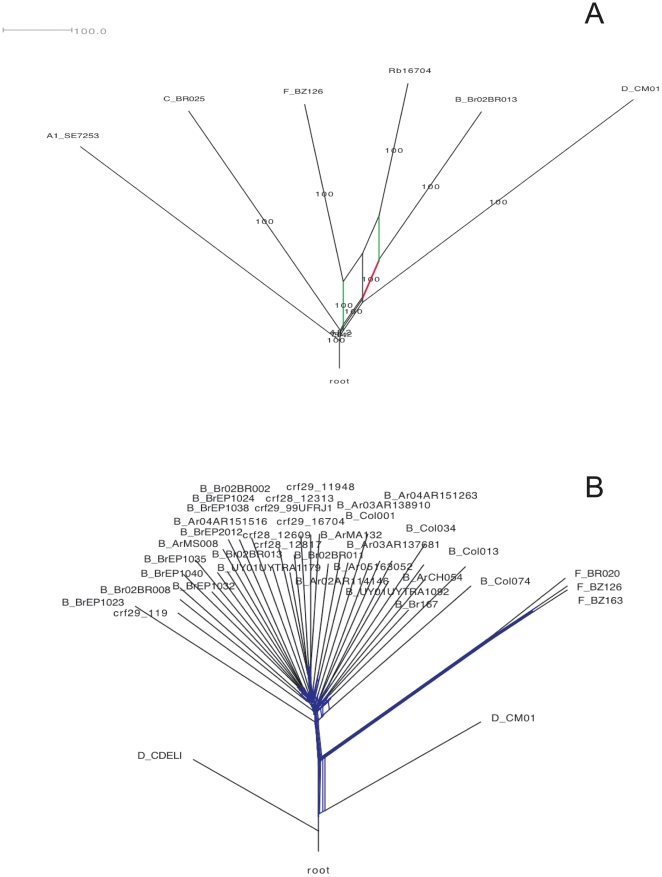
Phylogenetic network of HIV-1. Panel A depicts the network of the near full-length genome of a CRF_29 isolate (16704) and the conflicting relationship (green and red network) with its subtype B (Br02BR013) and subtype F (BZ126) lineages. Other references (subtype A, C, D) are also included. Numbers represent bootstrap support. Panel B depicts the network constructed with a recombination-free 3427-bp fragment and shows all isolates and CRFs branching off from the same node. The topology shows that there is no network between the distinct subtypes (subtypes A, B, C, D and F).

**Figure 5:**
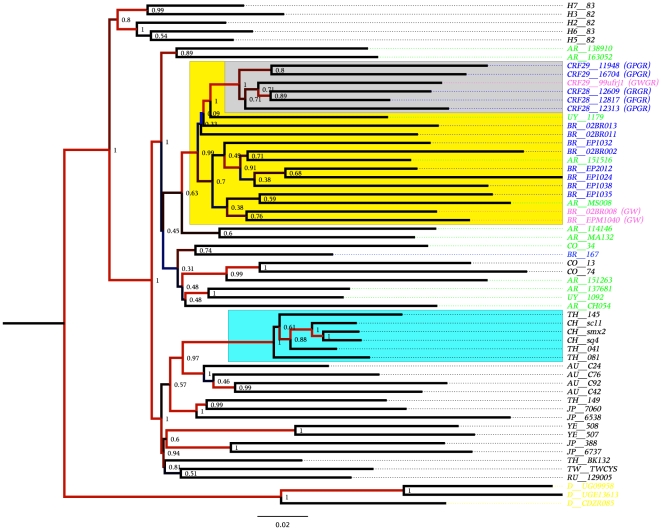
Bayesian tree constructed with a 3427-bp fragment free of recombinations to illustrate the common origin of all CRF_28 and CRF_29 isolates and other GWGR and GPGR Brazilian isolates. The yellow area delineates the cluster formed by the Brazilian isolates (blue letters). The grey area inside the Brazilian cluster denotes the group formed by CRF_28 and CRF_29. Sequences with GWGR at the V3 loop tetramer are indicated in pink letters in the tree. Argentinean isolates are indicated in green. Numbers in the nodes indicate the a posteriori support values. Subtype D sequences were used as outgroups in this tree (yellow letters).

In South America, according to the Los Alamos National Laboratory HIV database, more than 20% of HIV infections are from BF recombinant viruses. If these viruses recombine between two or more subtypes (parental subtype) and spread in the host population, they are designated as circulating recombinant forms (CRF). Trees constructed with non-recombinant DNA sequences of CRFs are monophyletic because all isolates share a common parental ancestor [18]. We used a variety of methods described elsewhere [19], [20] to select a recombination-free 3427-bp fragment from subtype B viruses to analyze the origin of CRF28 and CRF29. Initially, a window-based method was used to establish the mosaic pattern of the CRF28 and CRF29 near full-length genomes and then to select a non-recombinant fragment for the phylogenetic analysis. The mosaic pattern of three CRF28 (*viz*., 12609, 12817 and 12313) and three CRF29 (*viz*., 11948, 16704 and 99ufrj1) genomes are depicted in Figure 3. The genomic region after site 5.000 of HIV-1 is absent of recombination; this region is related to the parental subtype B lineage. To guarantee that the selected fragment was not a mosaic between two or more parental lineages, we performed further analysis using a phylogenetic network. The network of recombinant sequences is shown for illustrative purposes (Figure 4A); the conflicting phylogenetic relationships of this sequence are indicated by branches connecting both subtype B parental (Br02BR013) and subtype F parental lineages (BZ126). Next, a network was constructed with the recombination-free 3427-bp fragment where all CRF_BF plus subtype references were included (Figure 4). Since this network shows that there are not sequences in which the branches are connected by two distinct parental lineages, the selected fragment is not a chimera between distinct HIV-1 lineages. The Bayesian tree constructed with this 3423-bp fragment showed that all Brazilian isolates are within a cluster that has high support (yellow area, Figure 5). In addition, the CRF_BF isolates are also included in a subcluster that has high posterior probability (grey area in the tree). Remarkably, most CRF_BF isolates contain the GPGR motif at the V3 loop of the *env* gene, except for isolates CRF29_99ufrj1 (AY455778) with GWGR, CRF28_12609 (DQ085873) with GRGR and CRF28_12817 (DQ85874) with GFGR motif. It is interesting to mention that we found some isolates from Japan (*i.e*., AB286955, AB253430, AB221125) that are closely related to the CRF_BF28/29 (data not shown). We also found two BF recombinants (*i.e*., AY037267, AF408628) from Argentina with GWGR motif. These BF isolates are phylogenetically related with CRF12 (data not shown).

## Discussion

The HIV-1 subtype B epidemic in Brazil is unique because of the high frequency of isolates containing the GWGR tetramer at the V3 loop region. It has been suggested that GWGR is a distinct variant and less pathogenic than other subtype B isolates [17]. However, we showed that nearly all GWGR sequences isolated in Brazil are monophyletic and share a common ancestor with other subtype B isolates (GPGR, GGGR, GFGR, etc.) and it is unlikely that there are two distinct subtype B variants co-circulating in the Brazilian HIV-1 epidemic. In addition, the BF recombinants ARCH003 (AY037267) and AR063 (AF408628) from Argentina have GWGR tetramer; these sequences are related with CRF12_BF that has GPGR motif at the V3 loop. The CRF29_99ufrj1 (AY455778) has the GWGR tetramer, whereas others sequences isolated from this CRF have the GPGR tetramer. Our phylogenetic analysis showed that all CRF29 and CRF28 isolates share the same ancestral subtype B lineage regardless of the tetramer sequence at the V3 loop region (GWGR, GPGR, GRGR, GFGR, APGR). Thus, tryptophan replacement (*e.g*., GWGR-to-GxGR), which was previously observed in a within-host viral population [21], also occurs at the population level because all isolates of a specific CRF have a common origin and thus should have the same tetramer at the V3 loop.

HIV-1 infects mainly CD4-positive T cells and macrophages and the cellular tropism of HIV-1 is determined essentially by complex interactions between the viral envelope glycoprotein (Env) and cellular receptors. The *env* gene (V3 loop region) also determines whether the CCR5 or CXCR4 chemokine coreceptor will be used for entry [22], [23]. During the progress of HIV-1 infection, in roughly 50% of individuals the virus changes its chemokine receptor usage [24], [25]. Typically, in the early phase of infection the HIV-1 has a tropism for CCR5 (R5 variants), in the late phase of the infection viruses (X4 variants) that preferentially use CXCR4 emerge. At the late phase of HIV-1 infection, the within-host viral population may be composed exclusively by X4 variants, by variants capable of using both coreceptor (R5X4) or the viral population can be represent by equal amount of R5 and X4 variants [26], [27]. Alternatively, R5 and X4 viruses can even recombine in the course of within-host HIV-1 infection [28]. These variants have distinct properties the emergence of X4 variants is associated with a decline of CD4 cells and rapid disease progression [29]. Therefore, it has been suggested that X4 viruses might be more virulent than R5 viruses. R5 can be isolated preferentially from CD4+ memory T cells and X4 can be isolated from both memory and naïve cells [30], [31]. However, the reason for the change in the coreceptor usage and why it is restricted to a few patients remains uncertain. It has been suggested that R5 variants may have a selective advantage because of their higher replication rate in memory cells, compared with naïve cells [32], [33]. In addition, in resting CD4+ T cells the R5 variants, but not X4 variants, induce expression of genes involved in cell proliferation, this might facilitate the replication of R5 variants [34]. These findings might explain the predominance of R5 in the early infection, but not the later emergence of X4 variants. Recently, it has been shown that the turnover between naïve and memory cells might explain the switch from R5 to X4 variants in late infection [35]. It has been hypothesized that a more rapid increase on the rate of division of naïve cells observed with disease progression, despite the overall reduction in CD4 counts, provides a reasonable scenario for the overgrowth of X4 variants [35].

There must be a reason why X4 variants do not prevail in the early phase of HIV infection. Because the number of mutations associated with coreceptor changes is small and restricted to a limited region of V3 loop, X4 variants should increase their frequencies shortly after infection. It has been shown that in dendritic cells the production of R5 viral particle was higher than X4 [36], therefore favoring transmission of R5 variants through mucosal contact [37]. Nevertheless HIV transmission by the genital tract may independent of coreceptor usage [38] and R5 variants predominate in primary infection independently of the route of transmission [39].

The amino acid diversity of the V3 loop in Brazilian subtype B isolates suggests that there have been amino acid substitutions in the tetramer of this loop, which could be related to the HIV-1 preferences for coreceptor usage. There are high frequencies of GWGR and GFGR isolates detected in the Brazilian subtype B lineage. The 0.85 ratio of tryptophan + phenylalanine to proline (102+35/161) in the V3 loop of subtype B in Brazil cannot be explained by chance because of the disproportion in the tryptophan + phenylalanine to proline ratio observed in worldwide subtype B isolates (60+12/7689 = 0.009). Our previous work indicated that GWGR variants use exclusively CCR5 coreceptor [21]. Thus tryptophan and phenylalanine may provide an advantage to HIV-1 during the early stages of infection, and serve as a fitness advantage at the population level during new infection. The GWGR and GPGR sequences must have high adaptive fitness and distinct coreceptor preferences at the population level to be maintained at such high frequencies in Brazil.

Based on our previous study showing that tryptophan and phenylalanine in the V3 loop are related with coreceptor usage and our current results showing that almost all subtype B isolates sampled in Brazil share a common ancestor, we propose that the high frequency of tryptophan and phenylalanine is kept by a selective mechanism due to the distinct viral fitness in target cells.

## Materials and Methods

### Data sets

In total, 400 near full-length genomes (8160-bp genome fragment) of HIV-1 subtype B were obtained from the Los Alamos HIV Sequence Database (http://hiv-web.lanl.gov\).

### Sequence alignment

The sequences were initially aligned using ClustalX [40] and then sequences were manually aligned using the SE-AL program, version 2.0 (Department of Zoology, Oxford University; http://evolve.zoo.ox.ac.uk/software/). Alignments used in this study are available at http://www.biotorrents.net/.

### Recombination analysis

Recombinants and breakpoints were identified with Simplot v.2.5 (http://sray.med.som.jhmi.edu/RaySoft) using sequences from established HIV-1 subtypes as references. Recombination was checked by the Bootscan method [41] using neighbor-joining and Kimura (two-parameter) substitution models over a sliding window of 500 bp with 80-bp increments. Window trees were replicated 100 times to provide bootstrap support for permuted trees. In addition, we used a phylogenetic network to describe the relationship of the HIV sequences and to detect recombinant sequences. This method was implemented with the SplitsTree4 software [42].

### Phylogenetic inference

The GTR model in the PhyML software [43] was used to construct maximum likelihood (ML) trees. All trees were displayed and edited using the FigTree software (http://tree.bio.ed.ac.uk/software/figtree/).

The Bayesian tree was inferred using the MrBayes program, version 3.1.2 [44] with the GTR+ gamma correction model. We made two independent runs of 2×10^7^ generations; the initial 10% of these generations were discarded as burn-in, and the runs were sampled every 100th generation.

We also used a Bayesian Markov chain Monte Carlo (BMCMC) coalescent framework, implemented in the BEAST package [45], that uses DNA sequences to estimate ancestral genealogies using relaxed molecular clock models (lognormal) that have advantages over traditional clock models [46], [47]. Multiple runs were performed using HKY85 model with a gamma correction with a MCMC chain length of 2×10^7^ and 10% burn-in. The convergence of the Bayesian analysis was evaluated with TRACER and the sample of trees was summarized into a single topology using TreeAnnotator software (Department of Zoology, Oxford University [http://evolve.zoo.ox.ac.uk/software/]).

## References

[1] Buonaguro L, Tornesello ML, Buonaguro FM (2007). Human immunodeficiency virus type 1 subtype distribution in the worldwide epidemic: pathogenetic and therapeutic implications.. J Virol.

[2] Kuiken CL, Leitner T, Rodrigo AG, Learn GH (1999). HIV-1 subtyping.. Computational and evolutionary analysis of HIV molecular sequences.

[3] Peeters M, Delaporte E (1999). [Genetic diversity of HIV infection worldwide and its consequences].. Med Trop (Mars).

[4] Leal E, Janini M, Diaz RS (2007). Selective pressures of human immunodeficiency virus type 1 (HIV-1) during pediatric infection.. Infect Genet Evol.

[5] Walker PR, Pybus OG, Rambaut A, Holmes EC (2005). Comparative population dynamics of HIV-1 subtypes B and C: subtype-specific differences in patterns of epidemic growth.. Infect Genet Evol.

[6] Abecasis AB, Vandamme AM, Lemey P (2009). Quantifying differences in the tempo of human immunodeficiency virus type 1 subtype evolution.. J Virol.

[7] Martinez-Cajas JL, Pant-Pai N, Klein MB, Wainberg MA (2008). Role of genetic diversity amongst HIV-1 non-B subtypes in drug resistance: a systematic review of virologic and biochemical evidence.. AIDS Rev.

[8] Soto-Ramirez LE, Renjifo B, McLane MF, Marlink R, O'Hara C (1996). HIV-1 Langerhans' cell tropism associated with heterosexual transmission of HIV.. Science.

[9] Gilbert MT, Rambaut A, Wlasiuk G, Spira TJ, Pitchenik AE (2007). The emergence of HIV/AIDS in the Americas and beyond.. Proc Natl Acad Sci U S A.

[10] Kuiken CL, de Jong JJ, Baan E, Keulen W, Tersmette M (1992). Evolution of the V3 envelope domain in proviral sequences and isolates of human immunodeficiency virus type 1 during transition of the viral biological phenotype.. J Virol.

[11] Morgado MG, Sabino EC, Shpaer EG, Bongertz V, Brigido L (1994). V3 region polymorphisms in HIV-1 from Brazil: prevalence of subtype B strains divergent from North American/European prototype and detection of subtype F.. AIDS Res Hum Retroviruses.

[12] Potts KE, Kalish ML, Lott T, Orloff G, Luo CC (1993). Genetic heterogeneity of the V3 region of the HIV-1 envelope glycoprotein in Brazil. Brazilian Collaborative AIDS Research Group.. Aids.

[13] Taylor BS, Sobieszczyk ME, McCutchan FE, Hammer SM (2008). The challenge of HIV-1 subtype diversity.. N Engl J Med.

[14] Diaz RS, Leal E, Sanabani S, Sucupira MC, Tanuri A (2008). Selective regimes and evolutionary rates of HIV-1 subtype B V3 variants in the Brazilian epidemic.. Virology.

[15] Louwagie J, Delwart EL, Mullins JI, McCutchan FE, Eddy G (1994). Genetic analysis of HIV-1 isolates from Brazil reveals presence of two distinct genetic subtypes.. AIDS Res Hum Retroviruses.

[16] Ndung'u T, Renjifo B, Novitsky VA, McLane MF, Gaolekwe S (2000). Molecular cloning and biological characterization of full-length HIV-1 subtype C from Botswana.. Virology.

[17] Santoro-Lopes G, Harrison LH, Tavares MD, Xexeo A, Dos Santos AC (2000). HIV disease progression and V3 serotypes in Brazil: is B different from B-Br?. AIDS Res Hum Retroviruses.

[18] Abecasis AB, Lemey P, Vidal N, de Oliveira T, Peeters M (2007). Recombination confounds the early evolutionary history of human immunodeficiency virus type 1: subtype G is a circulating recombinant form.. J Virol.

[19] Martins Lde O, Leal E, Kishino H (2008). Phylogenetic detection of recombination with a Bayesian prior on the distance between trees.. PLoS One.

[20] Milne I, Wright F, Rowe G, Marshall DF, Husmeier D (2004). TOPALi: software for automatic identification of recombinant sequences within DNA multiple alignments.. Bioinformatics.

[21] Leal E, Silva WP, Sucupira MC, Janini LM, Diaz RS (2008). Molecular and structural characterization of HIV-1 subtype B Brazilian isolates with GWGR tetramer at the tip of the V3-loop.. Virology.

[22] Choe H, Farzan M, Sun Y, Sullivan N, Rollins B (1996). The beta-chemokine receptors CCR3 and CCR5 facilitate infection by primary HIV-1 isolates.. Cell.

[23] Cormier EG, Dragic T (2002). The crown and stem of the V3 loop play distinct roles in human immunodeficiency virus type 1 envelope glycoprotein interactions with the CCR5 coreceptor.. J Virol.

[24] Boyd MT, Simpson GR, Cann AJ, Johnson MA, Weiss RA (1993). A single amino acid substitution in the V1 loop of human immunodeficiency virus type 1 gp120 alters cellular tropism.. J Virol.

[25] Schuitemaker H, Koot M, Kootstra NA, Dercksen MW, de Goede RE (1992). Biological phenotype of human immunodeficiency virus type 1 clones at different stages of infection: progression of disease is associated with a shift from monocytotropic to T-cell-tropic virus population.. J Virol.

[26] Scarlatti G, Tresoldi E, Bjorndal A, Fredriksson R, Colognesi C (1997). In vivo evolution of HIV-1 co-receptor usage and sensitivity to chemokine-mediated suppression.. Nat Med.

[27] Shioda T, Levy JA, Cheng-Mayer C (1991). Macrophage and T cell-line tropisms of HIV-1 are determined by specific regions of the envelope gp120 gene.. Nature.

[28] van Rij RP, Worobey M, Visser JA, Schuitemaker H (2003). Evolution of R5 and X4 human immunodeficiency virus type 1 gag sequences in vivo: evidence for recombination.. Virology.

[29] Shankarappa R, Margolick JB, Gange SJ, Rodrigo AG, Upchurch D (1999). Consistent viral evolutionary changes associated with the progression of human immunodeficiency virus type 1 infection.. J Virol.

[30] Blaak H, van't Wout AB, Brouwer M, Hooibrink B, Hovenkamp E (2000). In vivo HIV-1 infection of CD45RA(+)CD4(+) T cells is established primarily by syncytium-inducing variants and correlates with the rate of CD4(+) T cell decline.. Proc Natl Acad Sci U S A.

[31] Koot M, Schellekens PT, Mulder JW, Lange JM, Roos MT (1993). Viral phenotype and T cell reactivity in human immunodeficiency virus type 1-infected asymptomatic men treated with zidovudine.. J Infect Dis.

[32] Bleul CC, Wu L, Hoxie JA, Springer TA, Mackay CR (1997). The HIV coreceptors CXCR4 and CCR5 are differentially expressed and regulated on human T lymphocytes.. Proc Natl Acad Sci U S A.

[33] Davenport MP, Zaunders JJ, Hazenberg MD, Schuitemaker H, van Rij RP (2002). Cell turnover and cell tropism in HIV-1 infection.. Trends Microbiol.

[34] da Silva J (2006). Site-specific amino acid frequency, fitness and the mutational landscape model of adaptation in human immunodeficiency virus type 1.. Genetics.

[35] Ribeiro RM, Hazenberg MD, Perelson AS, Davenport MP (2006). Naive and memory cell turnover as drivers of CCR5-to-CXCR4 tropism switch in human immunodeficiency virus type 1: implications for therapy.. J Virol.

[36] Vanham G, Penne L, Allemeersch H, Kestens L, Willems B (2000). Modeling HIV transfer between dendritic cells and T cells: importance of HIV phenotype, dendritic cell-T cell contact and T-cell activation.. Aids.

[37] Meng G, Wei X, Wu X, Sellers MT, Decker JM (2002). Primary intestinal epithelial cells selectively transfer R5 HIV-1 to CCR5+ cells.. Nat Med.

[38] Hladik F, Lentz G, Akridge RE, Peterson G, Kelley H (1999). Dendritic cell-T-cell interactions support coreceptor-independent human immunodeficiency virus type 1 transmission in the human genital tract.. J Virol.

[39] van't Wout AB, Kootstra NA, Mulder-Kampinga GA, Albrecht-van Lent N, Scherpbier HJ (1994). Macrophage-tropic variants initiate human immunodeficiency virus type 1 infection after sexual, parenteral, and vertical transmission.. J Clin Invest.

[40] Larkin MA, Blackshields G, Brown NP, Chenna R, McGettigan PA (2007). Clustal W and Clustal X version 2.0.. Bioinformatics.

[41] Lole KS, Bollinger RC, Paranjape RS, Gadkari D, Kulkarni SS (1999). Full-length human immunodeficiency virus type 1 genomes from subtype C-infected seroconverters in India, with evidence of intersubtype recombination.. J Virol.

[42] Huson DH, Bryant D (2006). Application of phylogenetic networks in evolutionary studies.. Mol Biol Evol.

[43] Guindon S, Gascuel O (2003). A simple, fast, and accurate algorithm to estimate large phylogenies by maximum likelihood.. Syst Biol.

[44] Huelsenbeck JP, Ronquist F (2001). MRBAYES: Bayesian inference of phylogenetic trees.. Bioinformatics.

[45] Drummond AJ, Rambaut A, Shapiro B, Pybus OG (2005). Bayesian coalescent inference of past population dynamics from molecular sequences.. Mol Biol Evol.

[46] Lepage T, Bryant D, Philippe H, Lartillot N (2007). A general comparison of relaxed molecular clock models.. Mol Biol Evol.

[47] Thorne JL, Kishino H, Painter IS (1998). Estimating the rate of evolution of the rate of molecular evolution.. Mol Biol Evol.

